# Characteristics and resolution of hypertension in obese African American bariatric cohort

**DOI:** 10.1038/s41598-021-81360-y

**Published:** 2021-01-18

**Authors:** Charu Gandotra, Motahar Basam, Ankit Mahajan, Julius Ngwa, Gezzer Ortega, Daniel Tran, Terrence M. Fullum, Zaki A. Sherif

**Affiliations:** 1 Department of Cardiology, Internal Medicine, Howard University Hospital, Washington, DC USA; 2 Department of Surgery, Kaiser Permanente Los Angeles Medical Center, Los Angeles, CA USA; 3 Department of Cardiology, Hartford Healthcare, Putnam, CT USA; 4 Department of Medicine, Howard University Hospital, Washington, DC USA; 5 Department of Surgery, Brigham and Women’s Hospital, Harvard Medical School, Boston, MA USA; 6 Department of Surgery, Howard University Hospital, Washington, DC USA; 7 Department of Biochemistry and Molecular Biology, College of Medicine, Howard University, Washington, DC 20059 USA

**Keywords:** Cardiology, Interventional cardiology

## Abstract

Weight reduction continues to be first-line therapy in the treatment of hypertension (HTN). However, the long-term effect of bariatric malabsorptive surgical techniques such as Roux-en-Y Gastric Bypass (RYGB) surgery in the management of hypertension (HTN) is less clear. African Americans (AA) are disproportionately affected by obesity and hypertension and have inconsistent outcomes after bariatric surgery (BS). Despite a plethora of bariatric literature, data about characteristics of a predominantly AA bariatric hypertensive cohort including hypertension in obese (HIO) are scarce and underreported. The aims of this study were, (1) to describe the preoperative clinical characteristics of HIO with respect to HTN status and age, and (2) to identify predictors of HTN resolution one year after RYGB surgery in an AA bariatric cohort enrolled at the Howard University Center for Wellness and Weight Loss Surgery (HUCWWS). In the review of 169 AA bariatric patients, the average BMI was 48.50 kg/m^2^ and the average age was 43.86 years. Obese hypertensive patients were older (46 years vs. 37.89 years; *p* < .0001); had higher prevalence of diabetes mellitus (DM, 43.09% vs. 10.87%; p < .0001) and dyslipidemia (38.2% vs. 13.04%; p 0.002). Hypertensive AA who were taking ≥ 2 antihypertensive medications prior to RYGB were 18 times less likely to experience HTN resolution compared to hypertensive AA taking 0–1 medications, who showed full or partial response. Also, HIO was less likely to resolve after RYGB surgery in patients who needed ≥ 2 antihypertensive medications prior to surgical intervention.

## Introduction

Obesity, which afflicts one third of the United States population, is defined as body mass index (BMI) of ˃30 kg/m^2^^1^. In 2017–2018, the prevalence of adult obesity in USA was 42.4%, an increase of 30.5% from 1999–2000^2^. The age-adjusted prevalence of severe obesity at 9.2% was higher in women than in men; whereas the overall prevalence of obesity was highest in non-Hispanic black women (56.9%) compared to Hispanic women (43.7%), non-Hispanic white women (39.8%), and non-Hispanic Asian women (17.2%)^2^. In fact, non-Hispanic black women had the highest percentage of obesity among all races and age groups since 2011^2^. This is also further complicated by the current COVID-19 pandemic in which BMI and hypertension (HTN) linked to obesity may be central to understanding the disproportionate number of African American COVID‐19 mortality^3,4^. The overall total health-care costs attributable to epidemic obesity and overweight are significant and are projected to double every decade^5,6^.

Obesity is a complex disease, and its pathophysiology is multifaceted. Growing evidence in basic science and clinical literature suggests that obesity is a disorder of the energy homeostasis system^7^. Beyond diet, the interplay between genetic and environmental factors along with socioeconomic status and unhealthy lifestyle can confer obesity pathogenesis^8,9^. How these disparate inputs interact with genetic, epigenetic and developmental factors that predispose to obesity is a critical question for future studies. Currently, it is known that genetic susceptibility, decreasing estrogen levels, medication use and physical inactivity contribute to midlife obesity in menopausal women^10–13^. In some cases, underlying causes may be gene mutations in leptin–melanocortin pathway, endocrine and psychiatric disorders^14,15^. The obesity pathophysiology further extends to renal hemodynamics that is indicative of renal fatty acid uptake and alterations in metabolic effects^16–20^. Also, a substantial segment of obese patients presents with obstructive sleep apnea (OSA)^21^. Both renal hemodynamics and OSA can also result in significant improvement following surgical weight reduction^21–23^.

Obesity is an independent risk factor for diabetes mellitus (DM), cardiovascular disease (CVD), chronic kidney disease (CKD) and hypertension (HTN), the latter being the most common obesity-related comorbidity^22,24–29^. Increased CVD is attributed to increased adrenergic (or sympathetic) activity in essential (aka primary) HTN, which leads to a drastic elevation in heart failure^30–32^.

The special case of hypertension in obese (HIO), which is difficult to clinically dissociate from HTN due to obesity, is multifactorial and not simple to distinguish from primary HTN, which has unknown secondary cause. This reflects the lack of a standard definition for hypertension in obesity. The predominantly known pathways linking obesity to HTN include increased mechanical load; sympathetic nervous system stimulation; salt and water retention; insulin resistance (IR) and even alterations in the gut hormone profile and neural activity^15,33–36^. Obese individuals usually present with HTN at a younger age compared to individuals with primary HTN. But even in early presentation, HIO is less likely to be adequately controlled and medical intervention for weight loss has failed to show consistent benefit in terms of its resolution^37,38^.

Surgical intervention such as bariatric surgery (BS) as shown in the Gateway Randomized Trial^39^, is currently the most effective treatment modality for morbid obesity and HIO^28,29^. Bariatric surgery also serves as a practical approach for blood pressure control, sustained weight loss, and prevention of CVDs in obese patients with HTN^39,40^. However, the long-term management of BS in isolated HTN and its association with race remain unclear. The data show that in various bariatric cohorts, the rate of HTN resolution at 1-year post BS ranges from 38.8 to 59.6%^28,29,41^. Prevalence of HTN is highest and its resolution lowest among African Americans (AA) undergoing BS compared to other racial and ethnic groups^42–44^. Income brackets and bariatric surgery access^45^ as well as outcomes including weight-loss and comorbidity resolution^46,47^ in states such as Michigan and Texas, for example, differ between blacks and whites where blacks have higher rate of 30-day complications and lower weight loss at 1 year following Roux-en-Y gastric bypass (RYGB) surgery. Among comorbidities, the Gateway Randomized Trial noted that recent research efforts on BS heavily focused on metabolic and DM resolution, but not on hypertension^39^. Several recent meta-analyses^48^ and similar studies related to post-RYGB surgeries have also suggested that race is an important determinant of weight loss and comorbidity remission^49–59^. In most studies^60^, AAs were relatively underrepresented and did not account for several other comorbidities related to metabolic syndrome in AA obese women^61–66^.

In our study, the participants underwent RYGB and were predominantly AA obese women. There is a relative paucity of data about the features of HIO in AA seeking BS and the predictors of its resolution after RYGB surgery. We conducted this retrospective study (1) to describe the preoperative clinical characteristics HIO with respect to HTN status and age; and (2) to identify predictors of HIO resolution 1 year after RYGB surgery in an AA bariatric cohort enrolled at the Howard University Center for Wellness and Weight Loss Surgery (HUCWWS). Data were collected by review of medical records maintained at the HUCWWS.

## Materials and methods

All experimental protocols, methods and ethical requirements were carried out in accordance with relevant guidelines and regulations of the Howard University Office of Regulatory Research Compliance (ORRC) and the Institutional Review Board Committee (HU-IRB), which approved the waiver (exemption) for informed patient consent and granted the approval for the medical chart review (IRB-16-CMED-30 and IRB-12-CMED-29) (HU-IRB) for this study. In this cohort study, the de-identified patient medical records that were maintained at HUCWWLS between 2007 and 2013 were reviewed. Patients with a body mass index (BMI) ≥ 40 kg/m^2^ or a BMI ≥ 35 kg/m^2^ with associated comorbidities; enrolled in the bariatric surgery program to undergo non-revision sleeve gastrectomy (SG) or Roux-en-Y Gastric Bypass (RYGB); and self-identified as African American were included in the study. Data about preoperative clinical characteristics, within 1 month prior to BS such as age, gender, BMI, blood pressure, heart rate, creatinine and comorbidities were collected. The diagnosis of HTN (≥ 140/90 mmHg), DM (≥ 126 mg/dl (7 mmol/L), hypercholesterolemia (> 200 mg/dl) and obstructive sleep apnea (OSA) was extracted from each patient’s active list of medical problems as entered by the treating physician.

Preoperative clinical characteristics (within 1 month prior to bariatric surgery) of obese AA with HTN were compared to obese AA without HTN (normotensives) in overall cohort; and then among two age groups of < 40 years and ≥ 40 years. Next, a subset of patients who underwent RYGB surgery and had one-year follow-up data available were analyzed. Within this subset, we identified patients who had no continued need for anti-hypertensive medications (responders) and patients who continued to need anti-hypertensive medications (non-responders) at 1 year after RYGB. Therefore, reduction in the number of medications was used as a criterion for a partial response to BS. Positive responders were then compared to non-responders in terms of their pre-operative baseline comorbidities, pre-operative medications and pre-operative pulse pressure to identify independent predictors of HTN resolution after RYGB surgery in African American bariatric patients.

We used descriptive statistics to assess patient baseline clinical and demographic factors associated with HTN. For categorical variables, we obtained the counts (proportions) and evaluated significant differences using chi-square or Fisher’s exact test. We performed Analysis of variance (ANOVA) for continuous variables to assess significance for any differences in means among subjects with/without HTN; Wilcoxon’s rank-sum test was applied for comparisons of non-normal continuously distributed data. We conducted a univariate analysis to assess potential confounders. We performed a multivariate logistic regression analyses to determine significant independent predictors of having HTN and HTN resolution. Variables that were significantly associated with HTN and HTN resolution were included in a multivariate logistic regression analysis. In the logistic regression, variables are presented as odds ratios and confidence intervals. P-values less than 0.05 were considered statistically significant and confidence intervals (CI) are calculated at the 95% level. Data analysis was conducted using the Statistical Analysis System software 9.3 (SAS Institute, Cary, NC) and Statistical Analysis and Graphics (NCSS 9.0.7, Kaysville, UT). Fisher Exact Test was implemented to calculate p-values for fatty liver contribution to HTN at pre-op and post-op events because some of the cells had values below 5.

## Results

### Clinical characteristics of HIO in the African American bariatric cohort

In this retrospective chart review of 169 African American bariatric patients, 86% were female, 67% were ≥ 40 years of age and 73% had HTN within 1 month of the planned bariatric intervention. The average BMI of the overall cohort was 48.50 kg/m^2^ and the average age was 43.86 years. Obese hypertensive patients were older (46 years vs. 37.89 years; p < 0.0001); had higher prevalence of DM (43.09% vs. 10.87%; p < 0.0001) and dyslipidemia (38.2% vs. 13.04%; p 0.002). BMI and prevalence of obstructive sleep apnea were not significantly different between the two groups. Table 1 compares the baseline characteristics of obese normotensive and obese hypertensive AAs enrolled to undergo non-revisional sleeve gastrectomy (n = 31) or RYGB (n = 138) surgery at HUCWWLS. Obese hypertensives were older (46 years vs. 37.89 years; *p* < 0.0001); had higher prevalence of DM (43.09% vs. 10.87%; *p* < 0.0001) and dyslipidemia (38.2% vs. 13.04%; *p* 0.002). BMI and prevalence of obstructive sleep apnea were not significantly different between the two groups. Multivariate analysis adjusting for baseline factors associated with HTN showed that obese hypertensive patients were more likely to be older and were 5 times more likely to have DM compared to obese normotensive patients (see Table 2).

**Table 1:** Baseline characteristics of obese normotensive versus obese hypertensive patients.

Characteristics	All participants (n = 169)	Obese normotensive (n = 46)	Obese hypertensive (n = 123)	P value
Age at time of surgery (years)	43.86 (10.42)	37.89 (11.23)	46.09 (9.20)	**< .0001**
BMI (kg/m^2^)	48.50 (8.25)	47.72 (7.77)	48.79 (8.44)	0.452
Gender (% female)	144 (86.23%)	40 (86.98%)	104 (85.95%)	0.866
Average household income ($)	84,563 (32,812)	82,972 (34,666)	85,165 (32,215)	0.704
**Procedure type**				
RYGB	135 (79.88%)	33 (71.74%)	102 (82.93%)	0.086*
SG	31 (18.34%)	13 (28.26%)	18 (14.63%)	
Surgery not performed	3 (1.78%)	0 (0.00%)	3 (2.44%)	
**Tobacco use**				
No	109 (90.83%)	31 (96.88%)	78 (88.64%)	0.284*
Yes	11 (9.17%)	1 (3.13%)	10 (11.36%)	
Systolic blood pressure	137.78 (19.30)	130.41 (14.87)	140.58 (20.09)	0.003
Diastolic blood pressure	83.16 (10.59)	80.89 (9.14)	84.03 (11.01)	0.094
Creatinine	0.86 (0.27)	0.80 (0.18)	0.88 (0.29)	0.167
**Degenerative joint disease**				
No	115 (68.45%)	31 (68.89%)	84 (68.29%)	0.941
Yes	53 (31.55%)	14 (31.11%)	39 (31.71%)	
**Hypercholesterolemia**				
No	116 (68.64%)	40 (86.96%)	76 (61.79%)	**0.002**
Yes	53 (31.36%)	6 (13.04%)	47 (38.21%)	
**Obstructive sleep apnea**				
No	74 (44.05%)	24 (53.33%)	50 (40.65%)	0.143
Yes	94 (55.95%)	21 (46.67%)	73 (59.35%)	
**Diabetes mellitus**				
No	111 (65.68%)	41 (89.13%)	70 (56.91%)	** < .0001**
Yes	58 (34.32%)	5 (10.87%)	53 (43.09%)	

BMI = Body Mass Index (Weight/Height^2^); Data are expressed as mean (standard deviation) if numerical variables, or as the number of patients and their percentages (%), if categorical; *Fisher Exact Test; Hypertension in obese: (1) Patient reported having hypertension and/or required medication at the time of screening; (2) the patient had a systolic blood level ≥ 140 mmHg and/or diastolic levels ≥ 90 mmHg.

**Table 2:** Multivariate analysis for entire cohort.

Factors	OR	95% Wald CL		P-value
Age	1.061	1.018	1.105	0.0046
Systolic blood pressure	1.034	1.009	1.059	0.0065
Diabetes mellitus	5.173	1.754	15.257	0.0029

OR = Odds Ratio; C.I. = Confidence Interval; Stepwise Logistic Regression Analysis to obtain OR.

This cohort was further divided into two age groups (< 40 years and ≥ 40 years) and the baseline clinical characteristics were compared between obese normotensive and obese hypertensive AA patients (Table 3). This subgroup analysis showed that in patients younger than 40 years, there was no significant relationship between DM and HTN; univariate analysis showed that obese hypertensive patients were about 5 years older (34.47 years versus 29.04 years with p-value 0.0004) and had approximately 10 mm Hg higher pulse pressure (53.38 mm Hg versus 43.79 mm Hg p-value 0.037) driven by significantly elevated systolic blood pressure, compared to the obese normotensive AA patients. These differences were not significant after multivariate analysis.

**Table 3:** Baseline characteristics of obese normotensive vs. obese hypertensive patients by age group.

Characteristics	All participants (n = 169)	Age < 40 (n = 56)			Age ≥ 40 (n = 113)		
		Normal (n = 24)	Pre-operative HTN (n = 32)	P value	Normal (n = 22)	Pre-Operative HTN (n = 91)	P value
Age at time of surgery (years)	43.86 (10.42)	29.04 (6.02)	34.47 (4.33)	**0.0004**	47.55 (6.60)	50.18 (6.58)	0.082
BMI (kg/m^2^)	48.50 (8.25)	49.07 (8.10)	50.79 (8.55)	0.363	46.25 (7.30)	48.09 (8.34)	0.355
Gender (% female)	144 (86.23%)	22 (91.67%)	28 (87.50%)	0.691	18 (81.82%)	76 (85.39%)	0.742
Average household income ($)	84,563 (32,812)	83,705.70 (39,483.93)	70, 074.40 (25,626.00)	0.335	82,204.73 (29,724.03)	90,251.61 (32,732.67)	0.403
**Procedure type**							
RNY (0)	135 (79.88%)	15 (62.50%)	27 (84.38%)	0.117	18 (81.82%)	75 (82.42%)	0.867
Sleeve (2)	31 (18.34%)	9 (37.50%)	5 (15.63%)		4 (18.18%)	13 (14.29%)	
Surgery not performed (4)	3 (1.78%)	0 (0.00%)	0 (0.00%)		0 (0.00%)	3 (3.30%)	
**Tobacco use**							
No	109 (90.83%)	15 (93.75%)	20 (80.00%)	0.376	16 (0.00%)	58 (92.06%)	0.577
Yes	11 (9.17%)	1 (6.25%)	5 (20.00%		0 (0.00%)	5 (7.94%)	
Pre-operative systolic blood pressure	137.78 (19.30)	129.23 (15.11)	140.48 (19.64)	**0.032**	131.59 (14.89)	140.61 (20.37)	0.062
Pre-operative diastolic blood pressure	83.16 (10.59)	81.45 (7.94)	85.39 (10.71)	0.223	80.32 (10.36)	83.53 (11.14)	0.213
Pre-operative pulse pressure	51.71 (18.56)	43.79 (17.92)	53.38 (17.61)	**0.037**	51.27 (9.59)	53.32 (20.31)	0.392
Pre-operative heart rate	80.50 (13.82)	76.86 (18.94)	83.97 (12.03)	0.136	77.33 (10.29)	80.96 (13.49)	0.196
Creatinine	0.86 (0.27)	0.81 (0.15)	0.84 (0.21)	0.905	0.79 (0.21)	0.89 (0.31)	0.110
**Pre-operative degenerative joint disease**							
No	115 (68.45%)	17 (70.83%)	25 (78.13%)	0.533	14 (66.67%)	59 (64.84%)	0.874
Yes	53 (31.55%)	7 (29.17%)	7 (21.88%)		7 (33.33%)	32 (35.16%)	
**Pre-operative hyper cholesterol**							
No	116 (68.64%)	22 (91.67%)	23 (71.88%)	0.093	18 (81.82%)	53 (58.24%)	0.050
Yes	53 (31.36%)	2 (8.33%)	9 (28.13%)		4 (18.18%)	38 (41.76%)	
**Pre-operative obstructive sleep apnea**							
No	74 (44.05%)	15 (62.50%)	14 (43.75%)	0.165	9 (42.86%)	36 (39.56%)	0.781
Yes	94 (55.95%)	9 (37.50%)	18 (56.25%)		12 (57.14%)	55 (60.44%)	
**Pre-operative diabetes**							
No	111 (65.68%)	22 (91.67%)	23 (71.88%)	0.093	19 (86.36%)	47 (51.65%)	**0.003**
Yes	58 (34.32%)	2 (8.33%)	9 (28.13%)		3 (13.64%)	44 (48.35%)	

BMI = Body Mass Index (Weight/ Height^2^); Data are expressed as mean (standard deviation) if numerical variables, or as the number of patients and their percentages (%), if categorical; * Fisher Exact Test; Pre-operative HTN: (1) Patient reported having hypertension and/or required medication at the time of screening; (2) the patient had a systolic blood level ≥ 130 mmHg and/or diastolic levels ≥ 90 mmHg.

Table 4 shows the number of participants with pre-operative hypertensive medications by age group (i.e. between < 40- and > 40-year-old groups). It is clear from the table that pre-operatively multiple medications are used more often and at a higher percentage by the > 40-year-old hypertensive patients than the younger age groups.

**Table 4:** Number of participants with pre-operative hypertensive medications by age group.

Pre-operative HTN Meds	Age < 40 (n = 56)		Age ≥ 40 (n = 113)	
	Frequency	Percent	Frequency	Percent
0	30	53.57	28	25.45
1	17	30.36	33	30.00
2	9	16.07	31	28.18
3	0	0.00	12	10.91
4	0	0.00	4	3.64
5	0	0.00	2	1.82

*3 participants with missing medication status.

In patients 40 years of age or older, obese hypertensive AA patients were 8 times more likely to have DM compared to obese normotensive AA patients irrespective of their BMI and this relationship-maintained significance even after multivariate analysis was performed (Table 5). Pre-operative pulse pressure was elevated in both obese normotensives and obese hypertensives with no significant between group difference (51.27 versus 53.32; p-value 0.392).

**Table 5:** Subgroup multivariate analysis.

Factors	Age < 40 years				Age ≥ 40 years			
	OR	95% Wald CL		P-value	OR	95% Wald CL		P-value
Body mass index	0.981	0.894	1.075	0.6772	1.066	0.973	1.169	0.1703
Systolic blood pressure	1.046	0.992	1.103	0.0974	1.052	0.999	1.108	0.0554
Diastolic blood pressure	0.982	0.895	1.077	0.6952	0.995	0.917	1.080	0.8997
Gender (ref = female)	3.010	0.105	86.567	0.5203	0.228	0.040	1.314	0.0981
Degenerative joint disease	0.486	0.077	3.085	0.4444	0.666	0.197	2.251	0.5126
Hypercholesterolemia	1.979	0.178	22.029	0.5787	4.335	0.930	20.202	0.0618
Obstructive sleep apnea	4.482	0.956	21.003	0.0570	0.605	0.160	2.291	0.4598
Diabetes mellitus	3.112	0.299	32.337	0.3419	8.263	1.725	39.586	**0.0082**

OR = Odds Ratio; C.I. = Confidence Interval.; Logistic Regression Analysis to obtain OR.

### Clinical characteristics associated with non-resolution of hypertension 1-year after RYGB surgery in the African American bariatric cohort

A subset of 133 patients who underwent RYGB was then evaluated. This group comprised predominantly of women (83%), with a mean age of 47 years and mean BMI of 49 kg/m^2^. Data about HTN status at 1-year after RYGB were available for only 57 patients. HTN remission rate at 1-year after RYGB surgery in this cohort was 49%. There were no significant differences in baseline clinical characteristics (Table 6) between positive responders and non-responders. However, multivariate logistic regression analysis showed that taking 0–1 antihypertensive medications and lower BMI pre-operatively were significantly associated with resolution of HTN after RYGB surgery in this African American bariatric cohort (Table 7). Hypertensive patients who were taking ≥ 2 antihypertensive medications pre-operatively were 18 times more likely to experience HTN non-resolution after RYGB surgery compared to hypertensive patients taking 0–1 medications. Thus, reduction in the number of medications can serve as a criterion for albeit partial response to RYGB-related surgical weight reduction.

**Table 6:** Characteristics of responders versus non-responders.

Baseline characteristics	All participants (n = 133)	Responders (n = 28)	Non-responders (n = 29)	P value
Age at time of surgery (years)	46.71 (9.31)	43.39 (10.16)	48.79 (7.72)	0.028
BMI (kg/m^2^)	48.90 (8.57)	47.86 (8.33)	52.29 (10.28)	0.080
Gender (% female)	63 (82.89%)	23 (78.79%)	29 (100.00%)	0.023*
Average household income ($)	83,304 (30,368)	76,390 (29,674)	79,352 (22,269)	0.673
BMI change (baseline vs. 1 year)	30.69 (6.97)	30.95 (6.22)	29.52 (6.99)	0.438
**Diabetes**				
No	67 (50.38%)	12 (42.86%)	15 (51.72%)	0.503
Yes	66 (49.62%)	16 (57.14%)	14 (48.28%)	
**Insulin**				
No	103 (79.23%)	20 (74.07%)	22 (75.86%)	0.877
Yes	27 (20.77%)	7 (25.93%)	7 (25.93%)	
**Degenerative joint disease**				
No	99 (74.44%)	21 (75.00%)	19 (65.52%)	0.434
Yes	34 (25.56%)	7 (25.00%)	10 (34.48%)	
**Hyper cholesterol**				
No	78 (58.65%)	15 (53.57%)	15 (51.72%)	0.889
Yes	55 (41.35%)	13 (46.43%)	14 (48.28%)	
**Obstructed sleep apnea**				
No	48 (36.09%)	10 (35.71%)	9 (31.03%)	0.708
Yes	85 (63.91%)	18 (64.29%)	20 (68.97%)	

BMI = Body Mass Index (Weight/Height^2^); Data are expressed as mean (standard deviation) if numerical variables, or as the number of patients and their percentages (%), if categorical; * Fisher Exact Test ; * Missing follow up data n = 76.

**Table 7:** Multivariate logistic regression analysis investigating association between clinical factors and hypertension non-resolution.

Factors	OR	95% C.I		P-value
Age at surgery	1.078	0.987	1.177	0.095
Pre-operative body mass index	1.104	1.005	1.212	0.039
Pre-operative hypertensive meds. (≥ 2 vs. 0 – 1)	18.574	4.188	82.367	**0.0001**

C.I. = Confidence Interval; OR = Odds Ratio.

## Discussion

In our study, the participants had a high average income ($86,000) and had access to good nutrition that were either absent or scarce to many AA obese women studied in the past. Also, the very rare obesity studies that were conducted among the AA population were not associated with HTN and type 2 DM that disproportionally affect life expectancy in AA women. In a retrospective study by Shah et al., that looked at 3795 RYGB-operated obese patients, prevalence of pre-operative HTN was 40%^67^ and in the study by Flores et al.^68^, the prevalence of pre-operative HTN was 50%. Our study examined an AA bariatric cohort and showed a much higher prevalence of pre-operative HTN of 73%; 57% in patients aged < 40 years and 80% in patients aged ≥ 40 years. This finding is in concert with the latest NHANES data (https://www.cdc.gov/nchs/data/factsheets/factsheet_nhanes.pdf) that showed higher prevalence of obesity and HTN in non-Hispanic Blacks in both young and middle-age groups^69^. But, this may also reflect that African American patients seek bariatric intervention at a higher comorbidity burden, perhaps due to different health behaviors.

Our study highlights the differences in the clinical characteristics of obese hypertensives versus obese normotensives overall and in relation to age in AA, who have been previously under-represented in bariatric studies. Both age and DM were significantly associated with HTN in our study, consistent with the Lilliam Flores et al. study^68^. Our analysis shows that in obese AA aged ≥ 40 years, the prevalence of DM is eight times more in patients with HTN versus without HTN regardless of the body mass index, which is likely related to the metabolic syndrome. But association between HTN and DM was not significant in younger patients aged < 40 years.

The co-occurrence of HTN, DM and obesity is referred to as metabolic syndrome (MetS) diagnosed when any of the following three out of five clinical risk factors are present^70^: impaired fasting serum glucose; low levels of serum high-density lipoprotein (HDL) cholesterol; elevated serum triglycerides (i.e. Hypertriglyceridemia); central obesity and HTN. The prevalence of metabolic syndrome increases with age and disproportionately affects AA^71–73^. This was seen in our bariatric cohort as well. Interestingly, despite comparable BMI, there was no increased prevalence of DM noted in the obese hypertensives compared to obese normotensives at age < 40 years. But, the prevalence of HTN increased from 57 to 80% and prevalence of DM increased from 28 to 48% after age 40 years, without notable change in BMI over time. This likely reflects the importance of duration of exposure to obesity and the related milieu in increasing the burden of cardiovascular comorbidities. Adipose tissue is not just a fat storage, but it is an endocrine organ. It secretes a variety of biologically active derivatives, such as angiotensinogen, adipokines, proinflammatory and inflammatory molecules (interleukin-1β, interleukin-6, tumor necrosis factor-α, C-reactive protein), reactive oxygen species, homeostasis modulating compounds and acute phase reaction proteins. This leads to a proinflammatory and prothrombotic state associated with vascular dysfunction leading to hypertension. In obese individuals, there is increase in circulating blood volume, increased heart rate, increased cardiac output, endothelial dysfunction and loss of arterial compliance (arterial stiffening), all contributing to HIO^34,35^.

Though our study is a small retrospective cohort study, it shows the interaction of age, HTN and DM in an obese African American bariatric cohort. Due to cross sectional nature, this study cannot establish temporal relationship between DM and HTN. Small sample size resulted in wide confidence intervals; nevertheless, there was a significant association between DM, age and HTN in African American bariatric patients. Waist circumference measurements were not available.

We also evaluated a small subset of patients who had one year follow up data available to identify clinical factors associated with resolution of HTN 1-year after bariatric surgery. As most of patients with available follow-up data had undergone RYGB, only this surgical cohort was analyzed. One year follow up rate was low at 43% and HTN resolution data was available only in 57 patients. This analysis showed that hypertensive patients who were taking ≥ 2 antihypertensive medications pre-operatively had a very high likelihood of non-resolution of hypertension one year after RYGB surgery. Poor follow up rate significantly limits conclusions from this analysis. However, the reduction observed in the number of medications post-RYGB surgery in our study can be used as a criterion for a recognizable response since it represents a less severe disease or even a well-controlled disease as exemplified in the Gateway Randomized Trials designed to assess the impact of BS in patients with obesity and hypertension^39^.

Also, information about other variables previously described to be associated with hypertension resolution such as duration of hypertension, percentage excess weight loss and pre-operative vitamin D levels was not available in the health records.

These findings contribute to our understanding of the bariatric African American cohort whose aging is associated with higher cardiovascular comorbidity burden. Also, obese African-American bariatric patients with pre-operative HTN who require none or at most 1 antihypertensive medication to control their blood pressure are much more likely to experience hypertension resolution at 1-year following bariatric surgery.

The limitations of this study include (1) retrospective nature, (2) small sample size, (3) lack of availability of anthropometric data other than BMI, (4) poor 1-year follow up rate, especially dietary habits, (5) lack of availability of excess body weight loss and (6) lack of generalizability.

For generalizability and addressing the limitations stated above, future longitudinal studies may be required to examine large multiethnic cohorts; further validation of the contribution of age and DM to HTN in obese individuals; evaluation of ethnic variations; refinement of the definition of “obesity related hypertension”; and the wholesome enhancement of treatment strategies aimed at HTN resolution in bariatric patients.

## Abbreviations

AA: African American
BMI: Body Mass Index
BS: Bariatric surgery
DM: Diabetes mellitus
HTN: Hypertension
HIO: Hypertension in obese
RYGB: Roux-en-Y gastric bypass
HUCWWS: Howard University Center for Wellness and Weight Loss Surgery
IR: Insulin resistance
MetS: Metabolic syndrome
NHB: Non-Hispanic Black
SG: Sleeve gastrectomy
